# An initiative to reduce psychiatric boarding in a Cape Town emergency department

**DOI:** 10.4102/sajpsychiatry.v29i0.2075

**Published:** 2023-11-14

**Authors:** Clint A. Hendrikse, Peter Hodkinson, Daniël J. van Hoving

**Affiliations:** 1 Division of Emergency Medicine, Faculty of Health Sciences, University of Cape Town, Cape Town, South Africa; 2 Emergency Department, Mitchells Plain Hospital, Cape Town, South Africa; 3 Division of Emergency Medicine, Faculty of Health Sciences, Stellenbosch University, Cape Town, South Africa

**Keywords:** psychiatric boarding, access block, emergency medicine, readmission rate, crowding

## Abstract

**Background:**

Psychiatric boarding in Emergency Departments (ED) is a global challenge which results in long ED length of stays (LOS) with significant consequences on patient care and staff safety.

**Aim:**

This study investigated the impact of an initiative to reduce psychiatric boarding on LOS and readmission rate, as well as explored the relationship between boarding times and LOS.

**Setting:**

This study was conducted at Mitchells Plain Hospital, a large district-level hospital in Cape Town.

**Methods:**

This cross-sectional study collected data for 24 months, which included a 9-month period prior to the initiative and 16 months thereafter. Data were collected retrospectively from official electronic patient registries. The initiative comprised of inpatient hallway boarding as a full-capacity protocol with the accompanying capacitation of psychiatric wards to accommodate the additional burden.

**Results:**

The initiative was associated with a decrease of 95% (*p* < 0.001) in boarding time, 13% (*p* < 0.001) in ward LOS and 25% (*p* < 0.001) in hospital LOS. Ward LOS were found to be independent of ED boarding times. The readmission rate increased from 12% to 18% post intervention.

**Conclusion:**

The initiative resulted in a sustainable improvement in boarding times and LOSs. The observational nature of this study precludes concrete conclusions and further investigations into psychiatric inpatient hallway boarding are recommended.

**Contribution:**

Inpatient hallway boarding could be a feasible option to reduce the risk. Psychiatric boarding times in the ED are independent of ward LOS, rendering it devoid from any value from a lean and economic perspective.

## Introduction

Hospitals and Emergency Department (ED) managers around the globe have described psychiatric boarding as the number one problem of their EDs.^1^ Despite there being no consensus regarding the definition, psychiatric boarding commonly refers to the time that mental and behaviourally disturbed patients spend waiting in an ED for an inpatient hospital bed or for transfer to another facility.^2^ Although boarding in general is a common challenge globally, patients with mentally and behavioural disturbances tend to be disproportionally affected with boarding times reported up to three times higher.^2,3^ This, together with the fact that they are 2.5 times as likely to require admission, results in very long ED length of stays (LOS) with significant consequences.^4^

Patients with mental and behavioural disorders do not receive high quality care while they are boarding in EDs and even though they are aware of the lack of resources, they perceive their treatment in the ED to not be a priority.^2,5^ Sixty per cent of ED directors in American hospitals report that no psychiatric services are provided during the boarding period, even though they require more nursing care than patients with no mental and behavioural disorders.^6^ Psychiatric boarding contributes to ED crowding which leads to increased morbidity and mortality for all ED patients.^7,8^ It consumes scarce ED resources, prolongs the time patients wait for potentially life-saving interventions, and reduces the number of treatment beds available to accommodate surges in demand.^9^

The burden of psychiatric boarding in EDs is likely to worsen as the prevalence of mental health conditions is increasing with a consequent increase in ED presentations.^4,10^ This is as a result of a wider mental healthcare delivery crisis and a failure of the outpatient care system.^11^ Most countries globally have followed a decentralisation strategy for some time, integrating mental healthcare into general health as the most cost-effective way to provide services.^12^ The situation on the ground however does not reflect the optimistic policies and programmes such that substantial gaps in service delivery remain in Africa.^12,13^

More than 80% of people with mental health disorders reside in low- and middle-income countries (LMICs) with mental illness and substance abuse disorders contributing to 8.8% and 16.6% of the total burden of disease respectively.^14^ Mood and psychotic disorders, together with alcohol abuse disorders represent nearly 20% of all disabilities related to health conditions in LMICs and people with a low socioeconomic status are eight times as likely to develop schizophrenia.^14^ The Western Cape province has the highest lifetime prevalence (39.4%) of mental health disorders in South Africa with the prevalence of anxiety, mood and substance disorders being 18.9%, 13.7% and 20.6%, respectively.^15^ Despite the significant contribution to the burden of disease and disability in Africa, mental and behavioural disorders generally enjoy less than 1% of already minuscule national health budgets.^16,17^ Although advances in mental healthcare in South Africa have been made, it is still regarded as a very low priority and consequently attains minimal resource allocation.^12,18^

Mitchells Plain Hospital, a public sector district-level hospital in Cape Town, South Africa, treats 55 000 patients per annum in the 25 bed ED, and serves a community of approximately 650 000 predominantly low- to middle-income residents. On 19 February 2018, the hospital embarked on an initiative to reduce psychiatric boarding in the ED by implementing two changes: (1) introducing inpatient hallway boarding as a full-capacity protocol (admitting all psychiatric boarders to the psychiatric ward regardless of bed availability); (2) capacitating the psychiatric ward to accommodate the additional burden. The aim of this study was to investigate the impact of this initiative on hospital and ward LOS. The first objective was to describe the burden and demographics of psychiatric boarders and the second objective was to explore the relationship between boarding times and LOS.

## Research methods and design

### Study design

This was a cross-sectional study, and data were collected retrospectively from official electronic patient registries. The premise for this study was conceived and conceptualised retrospectively.

### Study setting and description of the initiative

This study was conducted at Mitchells Plain Hospital, a large district-level hospital about 32 km from Cape Town’s central business district. It serves low-to middle-income communities of Mitchells Plain and mainly low-income communities of Philippi, a large nearby informal settlement. The ED is staffed by four specialist emergency physicians and is one of the busiest EDs in the Western Cape province, with an average of 4500 patients per month seeking emergency care with around 55% being of high acuity (triage category of red and orange).^19,20^ The psychiatric department is headed by a single psychiatrist and has a male ward within the hospital and a satellite female ward on the premises next to the hospital. Female patients are transported as interfacility transfers by emergency medical services (EMS) when admitted. Prior to the intervention, the average headcount of psychiatric boarders in the ED and adjacent Overnight Ward was ~30 at any given time. Psychiatric boarders were nursed in various clinical areas in the ED on mattresses on the floor, and the majority completed their 72-h observation in the ED. Even though additional nurses and security personnel were employed to oversee their care, patient and staff safety incidents were rife. Prior to the initiative, the on-site male psychiatric ward had 36 beds and the satellite female psychiatric ward had 14, with a bed occupancy rate of >95%. Together with the ED psychiatric boarders (~30), the total hospital burden of psychiatric patients at any given time was ~80.

On the day that the initiative was implemented, psychiatric boarders in the ED, together with the additional nurses and security personnel were moved to the psychiatric wards to be nursed there. Firstly, bed capacity was increased officially from 50 beds (36 male and 14 female) to 66 beds (48 male and 18 female) by utilising a different (bigger) satellite ward on the premises next to the hospital. Secondly, inpatient hallway boarding was implemented as a full-capacity protocol so that psychiatric boarders are moved to the ward as soon as they are referred, instead of waiting in the ED for a bed to become available. Admissions occurred regardless of bed availability, often requiring mattresses being utilised where physical space allowed, to create additional capacity. All admissions were managed by the psychiatric team and received the same package of care while occupying the hallway beds. This initiative was driven and supported by hospital management.

### Study population and sampling

All adult patients (≥18 years) that were referred to the psychiatric department from the ED between 01 June 2017 and 31 May 2019 (24 months) were eligible for inclusion. This convenience sample included 38 weeks (9 months) prior to the start of the initiative (19 February 2018). Only patients that were admitted to the psychiatric ward were included. Patients with incomplete clinical documentation, those who left before completion of hospital treatment, as well as those whose final diagnosis suggested a general medical condition as a cause for their symptoms were excluded from the study. Patients who were referred from a day hospital to the psychiatric department are fast tracked to the ward via the ED (direct admission) and were excluded as well.

### Data collection and management

Data were extracted from two electronic registries that collect routine administrative and clinical data. Process times, demographic details and diagnosis codes according to the International Statistical Classification of Diseases and Related Health Problems 10th Revision (ICD-10) were sourced from the Hospital and the Emergency Department Tracking and Information System (HECTIS), while ward discharge times were sourced from the hospital’s electronic patient management system, Clinicom. Both of these registries are official Western Cape Department of Health applications that prospectively collect routine data on all patients. The HECTIS also tracks patient movement through the ED and hospital, and is updated in real time. Data were de-identified once the data collection process was completed.

Process times were calculated in minutes and rounded to days or hours where appropriate. The definition of boarding for the purpose of this project was defined as waiting in the ED > 6 h for an inpatient bed after being referred to the psychiatric department.^21,22^ Boarding categories were adopted from Singer et al.^7^: 6–12 h; 12–24 h, 24–72 h and > 72 h. The readmission rate was defined by provincial policy as the number of patients with mental and behavioural disturbances who are re-admitted for any reason within 90 days of discharge from hospital.

### Data analysis

Categorical data were described with descriptive statistics and presented as frequency or percentages and non-random associations were assessed with the Chi^2^ test. The Kolmogorov–Smirnov test was used to test the distribution of continuous variables, and non-normal variables were described using median and percentiles (25% to 75%) and means compared with the Mann–Whitney *U* test. The Spearman’s correlation coefficient was used to determine the strength and direction of the association between boarding time and LOS; no outliers were removed. A post-hoc power calculation for the primary outcome (hospital LOS) comparing the means and standard deviation (s.d.) before and after the initiative, with an alpha = 0.05 resulted in a power of 100%. Statistical significance was defined as *p* < 0.05 and a clinically significant difference in ward- or hospital LOS was defined as 1 day (24 h).

### Ethical considerations

Ethical approval was obtained from the University of Cape Town Human Research Ethics Committee: HREC REF 539/2019 and facility approval was granted via the National Health Research Database: WC_201908_037. There was no patient participation, and a waiver of consent was approved.

## Results

**FIGURE 1:**
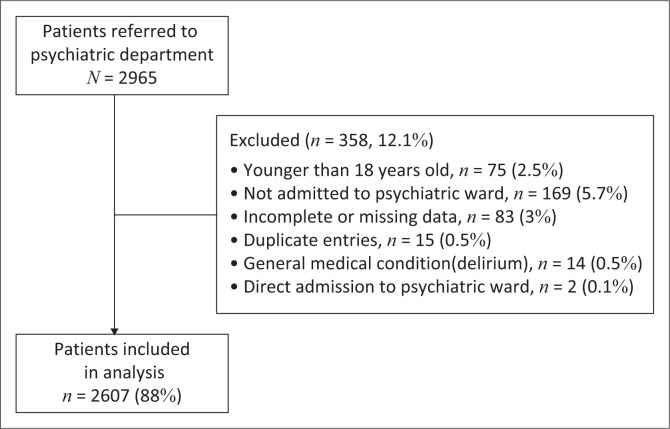
Flowchart of study population.

Overall, 97 357 patients presented to the ED during the study period of which 74 459 (76%) were adults. A total of 2965 (3%) patients were referred to the psychiatric department and were therefore eligible for inclusion. After applying exclusions, 2607 (88%) of patients were included in the final analysis (Figure 1).

The median age for male and female patients was 30 years (26–44) and 35 years (18–52), respectively. The demographic and clinical differences of the sample before and after the start of the initiative are presented in Table 1. Age (*p* = 0.337), gender (*p* = 0.214), triage category (*p* = 0.968), and day of arrival to the hospital (*p* = 0.184) were similarly distributed between the two groups. More patients arrived between 08:00 am and 16:00 pm after the start of the initiative (*p* = 0.009) but less patients had their decision to admit made between 08:00 am and 16:00 pm (*p* < 0.001). Patients with behavioural disturbances because of substance use were more prevalent after the initiative (5% vs 9%, *p* < 0.001), while patients with schizophrenia and other delusional disorders were more prevalent before the initiative (76% vs 71%, *p* < 0.001).

**TABLE 1:** Patient demographics and clinical characteristics of psychiatric admissions before and after the start of the initiative (*n* = 2607).

Variable	Total (*N* = 2607)†		Before (*N* = 809)‡		After (*N* = 1798)§		*P*
	*n*	%	*n*	31%	*n*	69%	
**Gender**							0.214
Male	1829	70	581	72	1248	69	-
Female	778	30	228	28	550	31	-
**Age (years)**							0.337
18–25	591	23	170	21	421	23	-
26–35	1031	40	310	38	721	40	-
36–45	524	20	178	22	346	19	-
46–55	255	10	83	10	172	10	-
56–65	161	6	53	7	108	6	-
66–75	38	2	11	1	27	2	-
> 75	7	0.3	4	0.5	3	0.2	-
**ICD-10 category**							< 0.001
F00–F09: Organic, including symptomatic mental disorders	23	1	9	1	14	1	-
F10–F19: Mental and behavioural disorders due to substance use	201	8	37	5	164	9*	-
F20–F29: Schizophrenia, schizotypal and delusional disorders	1888	72	616	76*	1272	71	-
F30–F39: Mood disorders	346	13	98	12	248	14	-
F40–F49: Neurotic, stress-related and somatoform disorders	20	1	2	0.2	18	1*	-
F60–F69: Disorders of adult personality and behaviour	3	0.1	3	0.4	0	0	-
F70–F79: Mental retardation	8	0.3	1	0.1	7	0.4	-
Other	118	4.5	43	5	75	4	-
**Day of arrival**							0.184
Monday	351	14	101	13	250	14	-
Tuesday	431	17	155	19*	276	15	-
Wednesday	419	16	117	15	302	17	-
Thursday	457	18	149	18	308	17	-
Friday	364	14	113	14	251	14	-
Saturday	308	12	89	11	219	12	-
Sunday	277	11	85	11	192	11	-
Weekday	2022	78	635	79	1387	77	0.444
Weekend	585	22	174	22	411	23	-
**Time of arrival**							0.009
08:00–16:00	999	38	280	35	719	40*	-
16:00–08:00	1608	62	529	65*	1097	60	-
**Time of disposition decision**							< 0.001
08:00–16:00	1101	42	420	52*	681	38	-
16:00–08:00	1506	58	389	48	1117	62*	-

† , admissions per week (mean = 25; s.d. = 7.1);

‡ , admissions per week (mean = 21 s.d. = 4.8);

§ , admissions per week (mean = 28. s.d. = 7.2).

* , Statistically higher proportion (*p* < 0.005).

**FIGURE 2:**
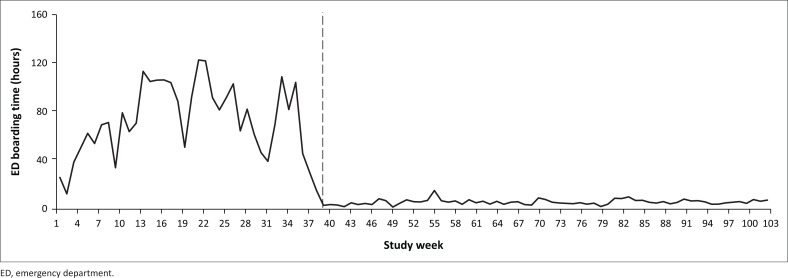
Median Emergency Department psychiatric boarding time per study week before and after the start of the initiative (week 39).

Figure 2 displays the median ED boarding time per week before and after the initiative was implemented. A significant and sustained reduction in ED boarding times after the initiative (study week 39) occurred.

A significantly higher proportion of patients boarded in the ED prior to the start of the initiative (90% vs 33%, *p* < 0.001), especially those who waited for more than 24 h (34% vs 4%, *p* < 0.001) (Table 2). The initiative was associated with a decrease of 95% (56 vs 3 h, *p* < 0.001) in psychiatric boarding times, 13% (16 vs 14 days, *p* < 0.001) in ward LOS and 25% (20 vs 15 days, *p* < 0.001) in hospital LOS. There was a statistically significant increase in the readmission rate of 6% (*p* < 0.001). Participants who boarded in the ED during the study period had a similar ward LOS compared to those who did not board (353 vs 352 h, *p* = 0.919), but experienced a significantly longer hospital LOS (429 vs 364 h, *p* < 0.001). The correlation between ED boarding time and ward LOS was non-linear with *R*_s_ = 0.022 (*p* = 0.27).

**TABLE 2:** Patient process times, boarding status, and throughput metrics of psychiatric admissions before and after the start of the initiative (*n* = 2607).

Variable	Total (*N* = 2607)		Before (*N* = 809)		After (*N* = 1798)		*P*
	*n*	%	*n*	31%	*n*	69%	
**Boarding category (hours)†**							< 0.001
None	1298	50	85	11	1213	67*	-
6–12	340	13	36	4	304	17*	-
12–24	296	11	81	10	215	12	-
24–72	339	13	274	34*	65	4	-
>72	334	13	333	41*	1	0.1	-
**Boarded in ED**							< 0.001
Yes	1309	50	724	90*	585	33	-
No	1298	50	85	11	1213	67*	-
**Readmitted**							< 0.001
Yes	409	16	94	12	315	18*	-
No	2198	84	715	88*	1483	83	-
**Median (Q_1_–Q_3_)**							
**Process times**							
ED boarding time (hours)	6	1–26	56*	24–104	3	1–8	< 0.001
ED length of stay (hours)	19	9–47	81*	46–128	13	7–23	< 0.001
Ward length of stay (days)	15	7–25	16*	8–27	14	7–24	0.001
Hospital length of stay (days)	16	9–27	20*	13–31	15	8–25	< 0.001
**Hospital length of stay (hours)**							
Boarding	429	233–672	488*	307–752	357	181–574	< 0.001
No boarding	364	189–605	386	255–745	361	188–600	0.051
**Ward length of stay (hours)**							
Boarding	353	170–616	378*	189–650	335	155–550	0.001
No boarding	352	174–597	368	222–722	351	173–586	0.126

ED, emergency department.

* , Statistically higher proportion or mean (*p* < 0.05).

† , Boarding is defined as waiting in the ED > 6 h for an inpatient bed after being referred to the psychiatric department.

**FIGURE 3:**
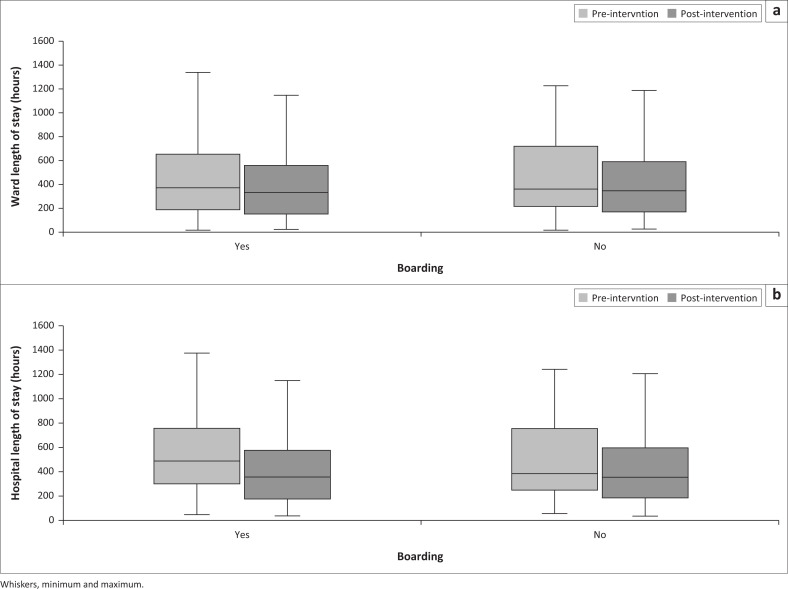
Clustered boxplots of (a) ward- and (b) hospital length of stay per boarding status pre- and post-intervention, *N* = 2706.

**FIGURE 4:**
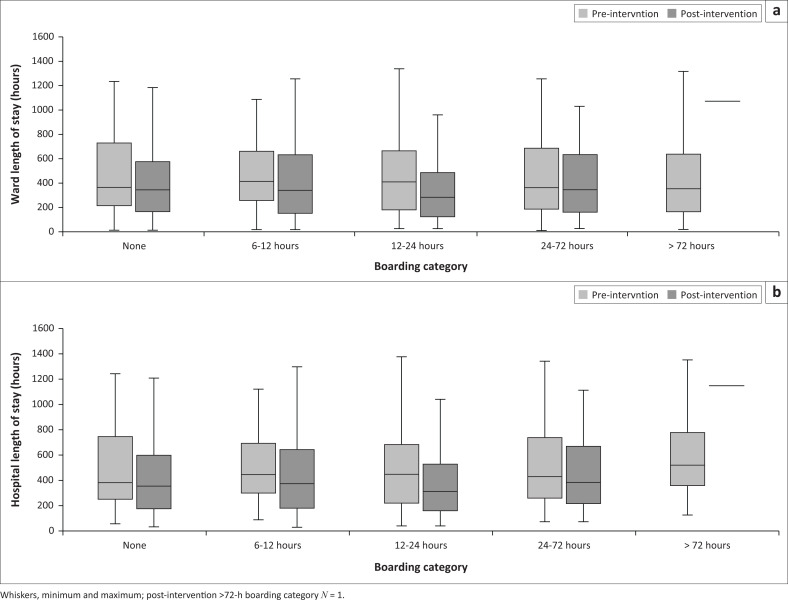
Clustered boxplots of (a) ward- and (b) hospital length of stay per boarding category pre- and post-intervention, *N* = 2706.

Figure 3 depicts ward- and hospital LOS for boarding vs no boarding, before and after the initiative. The median ward- and hospital LOS were universally shorter after the start of the initiative, whether boarding was present or not. After the start of the initiative, the median ward- and hospital LOS were statistically similar (335 vs 351 h, *p* = 0.077 and 357 vs 361 h, *p* = 0.476, respectively). Figure 4 depicts ward- and hospital LOS for each boarding category, before and after the initiative. The median ward- and hospital LOS was shorter for all categories of boarding after the start of the initiative, with the biggest difference in the 12 h–24 h boarding category. The median hospital LOS for the > 72 h-category was the longest, and disproportionally longer than the corresponding ward LOS.

## Discussion

The implementation of inpatient hallway boarding as a full-capacity protocol and the accompanying capacitation of psychiatric wards to accommodate the additional burden were associated with a decrease in the prevalence of psychiatric boarding in the ED by 57%. The median ED boarding time subsequently decreased by 53 h, the median ward LOS decreased by 2 days and the median hospital LOS by 5 days. This reduction was sustained over time, but a 6% increase in the readmission rate was noted.

Nine in 10 patients with mental and behavioural disorders boarded in the ED before the start of the initiative. This is substantially higher than the prevalence reported in high-income countries, although it varies considerably. In 2008, a survey of 328 EDs in the United States of America (US) indicated that 79% of patients with mental health emergencies boarded in the ED.^6^ In the same year, Nolan et al.^21^ assessed a cohort of nearly 35 000 ED patients across the US and found 22% of patients with mental and behavioural disorders board in EDs. An ED in Florida reported the prevalence to be 40% between 2010 and 2013, which increased to 70% in patients who were transferred to a different health facility for admission.^22^ Data from our study showed that female mental health users boarded for 6 h longer than their male counterparts, most likely because of the fact that they required transport to a different facility for admission. The paucity of data in LMICs precludes any concrete comparisons; therefore, a comprehensive nationwide evaluation of the burden of psychiatric boarding in South Africa and Africa is needed.

Hospital LOS is a quality metric that is often used to reflect efficiency within a hospital and a reduction is associated with improved bed turnover, allowing capacity and demand to be matched dynamically.^23^ An increase in hospital LOS results in ED crowding and is associated with worse outcomes and an increase in mortality.^23^ Hospital LOSs were universally and significantly shorter for all categories of boarding, after the initiative was implemented. This contradicts the findings of a recent study investigating more than 19 000 psychiatric admissions in Canadian EDs where psychiatric boarding was associated with a minimal increase in hospital LOS (14 min more in the hospital for a patient who boarded for ≥ 24 h, or 29 min more in the hospital for a patient who boarded for ≥ 72 h).^24^ This is unexpected considering that the reported median boarding time of 6.5 h is similar to the 6 h in this study. Patients in this study who boarded for ≥ 24 h had a 121-h increased hospital stay, while those who boarded for ≥ 72 h had a 147 h increase. This could be explained by the fact that both the ward and ED LOSs were much longer.

Ward LOS was similar for all categories of boarding, both before and after the initiative. The data indicate that the ward LOS of patients with mental and behavioural disturbances is independent of their ED boarding times. This access block-effect refers to a paradox where an increase in the duration of ED boarding times is associated with longer inpatient LOSs.^25^ Logically, the opposite is expected as one would assume that the *healing process* initiates in the ED with continuation of care in the ward – therefore expecting shorter ward stays for those with longer ED stays. This association however does not necessarily equal causation, as a reverse theory could also be plausible – a decrease in ward LOS leading to a decrease in ED boarding times. From a lean and economic perspective, the results of this study suggest that psychiatric boarding is devoid of any value. This study is however an observational study and further inferences will require an impact study.

The reduction in LOS came at a cost in the form of a higher readmission rate. The 6% increase in readmission rate may be as a result of premature discharges to accommodate the burden of admissions (perhaps now more visible and acutely felt by psychiatrists as opposed to when boarded in the ED). The increase should however be interpreted along with the increase in admissions per week from 21 prior to the initiative to 28 thereafter (Table 1). The readmission rate as a standalone quality metric does not necessarily refute the significant improvement in hospital and ED LOSs as a result of this initiative. Pre- and post-discharge factors associated with readmission, including: (1) individual vulnerability; (2) aftercare related factors; (3) community care and service responsiveness and (4) contextual factors and social support, should be assessed to understand the true impact.^26,27^

Even though the initiative was associated with a significant decrease in overall LOS, a comprehensive impact assessment was not performed and aspects like patient and staff perceptions about inpatient hallway boarding were not explored. Inpatient hallway boarding, as an alternative to boarding in the ED, is a strategy that has been used as a full-capacity protocol to distribute admitted patients to inpatient wards, to reduce ED crowding and improve overall patient flow.^28^ Surveys elsewhere that explored patients perceptions and experiences suggest that they overwhelmingly prefer inpatient hallway boarding to ED boarding.^28,29,30^ Shoham et al.^31^ found that inpatient hallway boarding in a hospital in Jerusalem lowered inpatient mortality (OR 0.76, [CI, 0.65 to 0.90]) but increased the 30-day readmission rate (OR, 1.18 [CI, 1.00 to 1.40]). Nursing perceptions differed depending on where they work, with unsurprisingly ward nurses being opposed to inpatient hallway boarding and ED or ex-ED nurses supporting it.^32^

### Strengths and limitations

This study investigated a large sample of nearly 3000 patients and therefore minimised the likelihood that random error could have impacted the results. The robust electronic patient registries ensured accurate data. A few potential confounders were not analysed including the time delay to the first consultation by a psychiatrist, the type of admission (index presentation vs known mental health user or voluntary vs assisted admission) and final (discharge) diagnosis. Readmissions to other facilities were not actively sought and a few cases could have been missed both prior and after the start of the initiative. An increase in readmissions in our hospital, with a concurrent decrease in readmissions elsewhere, may also indicate increased patient satisfaction by choosing to re-present at this hospital – this was however not explored. Operational practices within the psychiatric ward could have changed to adopt to the increased patient load – this was not explored in this study, but does require further investigation.

### Suggestions for future research

A follow-up study should determine whether the initiative and outcomes are sustainable. Future research should aim to perform a multicentre analysis and include potential confounders. Perceptions of patients and their families, as well as the nursing staff should be explored qualitatively with regards to inpatient hallway boarding. The impact of strategies that the psychiatric department utilised to manage the increased patient burden, as well as the effect of inpatient hallway boarding on the LOS of non-psychiatric patients, should be investigated.

## Conclusion

This study demonstrated a significant improvement in hospital LOS and ED boarding times after inpatient hallway boarding was implemented as a full-capacity protocol, together with the accompanying capacitation of psychiatric wards to accommodate the additional burden. This initiative halved the prevalence of psychiatric boarders and significantly decreased hospital LOS. Ward LOS was found to be independent of EC boarding times which from a lean- and economic perspective, suggests that psychiatric boarding is devoid of any value. The benefits of this initiative should be weighed up against the subsequent higher readmission rate. The observational nature of this study precludes concrete conclusions and further investigations into psychiatric inpatient hallway boarding are recommended.
